# Exploring the interplay between antiretroviral therapy and the gut-oral microbiome axis in people living with HIV

**DOI:** 10.1038/s41598-024-68479-4

**Published:** 2024-08-01

**Authors:** Aswathy Narayanan, Oscar Kieri, Jan Vesterbacka, Lokeshwaran Manoharan, Puran Chen, Mahin Ghorbani, Hans-Gustaf Ljunggren, Margaret Sällberg Chen, Soo Aleman, Anders Sönnerborg, Shilpa Ray, Piotr Nowak

**Affiliations:** 1 Department of Medicine Huddinge, Division of Infectious Diseases, Karolinska Institutet, Stockholm, Sweden; 2 Department of Infectious Diseases, Karolinska University Hospital, Stockholm, Sweden; 3 Department of Laboratory Medicine, National Bioinformatics Infrastructure Sweden (NBIS), SciLife, Lund University, Lund, Sweden; 4 Department of Medicine Huddinge, Center for Infectious Medicine, Karolinska Institutet, Stockholm, Sweden; 5 Department of Dental Medicine, Karolinska Institutet, Stockholm, Sweden; 6 Department of Laboratory Medicine, Division of Clinical Microbiology, ANA Futura, Karolinska Institutet, 141 52 Stockholm, Sweden; 7 Department of Laboratory Medicine, Division of Pathology, ANA Futura, Karolinska Institutet, 141 52 Stockholm, Sweden

**Keywords:** Gut microbiota, Oral microbiome, People living with HIV (PLWH), Integrase inhibitors (INSTIs), Dolutegravir (DTG), BMI, Data processing, Genetic databases, HIV infections, Bacteria, Microbial communities

## Abstract

The gut and oral microbiome is altered in people living with HIV (PLWH). While antiretroviral treatment (ART) is pivotal in restoring immune function in PLWH, several studies have identified an association between specific antiretrovirals, particularly integrase inhibitors (INSTI), and weight gain. In our study, we explored the differences in the oral and gut microbiota of PLWH under different ART regimens, and its correlation to Body Mass Index (BMI). Fecal and salivary samples were collected from PLWH (n = 69) and healthy controls (HC, n = 80). We performed taxonomy analysis to determine the microbial composition and relationship between microbial abundance and ART regimens, BMI, CD4^+^T-cell count, CD4/CD8 ratio, and ART duration. PLWH showed significantly lower richness compared to HC in both the oral and gut environment. The gut microbiome composition of INSTI-treated individuals was enriched with *Faecalibacterium* and *Bifidobacterium*, whereas non-nucleotide reverse transcriptase inhibitor (NNRTI)-treated individuals were enriched with *Gordonibacter*, *Megasphaera,* and *Staphylococcus*. In the oral microenvironment, *Veillonella* was significantly more abundant in INSTI-treated individuals and *Fusobacterium and Alloprevotella* in the NNRTI-treated individuals. Furthermore, *Bifidobacterium and Dorea* were enriched in gut milieu of PLWH with high BMI. Collectively, our findings identify distinct microbial profiles, which are associated with different ART regimens and BMI in PLWH on successful ART, thereby highlighting significant effects of specific antiretrovirals on the microbiome.

## Introduction

An estimated 29 million people living with HIV (PLWH) globally are receiving antiretroviral therapy (ART)^1^. ART usually consists of a combination of two nucleotide/nucleoside reverse transcriptase inhibitors (NRTIs) and either an integrase strand transfer inhibitor (INSTI), a non-nucleotide reverse transcriptase inhibitor (NNRTI), or a protease inhibitor (PI). Currently, INSTIs are the first-line treatment regimen according to WHO and national guidelines^2^. The introduction of ART has reduced HIV transmission and HIV-associated mortality increasing life expectancy of PLWH globally^3^. Several studies have established that both untreated and treated HIV infection is associated with increased inflammation, microbial translocation, and gut dysbiosis^4^. This is particularly relevant given the important role that microbiome plays in sustaining human health^5^. In fact, the bacteria inhabiting both the oral and gut microbiomes have the capacity to engage with the immune system, thereby facilitating its function. This is further corroborated by the observed shifts in the microbiome in a variety of disease states and chronic inflammatory conditions^6,7^. For instance, both gut and oral dysbiosis have been linked to obesity, as evidenced in laboratory investigations and patient cohorts^8–10^. We and others have previously reported that different ART regimens could have distinct modulating effects on the gut microbiome due to the anti-microbial properties of specific antivirals^5,11,12^. However, it is not fully understood how the different categories of antiretrovirals (ARV) modulate the microbiota of PLWH over time and whether newer ARVs have similar effects on the microbiome^11,13,14^. Earlier studies have shown that HIV infection disrupts the immune system, affecting both the oral and gut microbiomes^3,6^. Chronic inflammation resulting from HIV infection can promote the development of pathogens in the oral cavity, leading to conditions such as oropharyngeal candidiasis and periodontal diseases^15^. Even after the initiation of ART, inflammation in the oral cavity persists^6,16^, which further aggravates immune activation. Therefore, investigating the interplay between gut and oral microbiome changes is crucial for understanding immune dysregulation in PLWH. Given that dysbiosis in both the oral and gut microbiomes has been rarely investigated in PLWH, our study is of significant importance^3,6,16^.

Additionally, HIV infection is commonly associated with metabolic alterations, particularly in lipid metabolism and related hormones. These include increased triglycerides and LDL cholesterol, decreased HDL cholesterol, and insulin resistance, which contributes to elevated risk of cardiovascular diseases^17,18^. Furthermore, several studies have observed significant increased weight in PLWH on ART and weight-gain has been associated with certain class of antivirals like INSTI, particularly in the study groups, according to sex and ethnicity^19,20^. However, there is a knowledge gap whether the weight gain associated with ARV is mediated by the changes in microbiome.

In the current study, we have described the oral and gut microbiome in healthy controls (HC) and PLWH. Additionally, we have investigated the effect of different ART regimens, with focus on INSTI, on the gut microbiome of PLWH and their association with the Body Mass Index (BMI).

## Methods

### Study cohort

The study was part of an open-label, non-randomized clinical trial at the Karolinska University Hospital, Stockholm, Sweden, which investigated the safety and clinical efficacy of the mRNA BNT162b2 vaccine (Comirnaty^®^, Pfizer/BioNTech)^12^. The study was conducted according to the guidelines of the Declaration of Helsinki, and all participants provided written informed consent. The ethical permit was granted by the Swedish Ethical Review Authority (ID 2021-00,451, ID 2023-05,153-02). Fecal and oral samples were collected from 90 PLWH and 90 HC at the time of first vaccine dose. Individuals with antibiotic treatment three months before vaccination were excluded from analysis (PLWH: n = 21; HC: n = 10). The fecal and saliva samples were collected in RNA/DNA shield (Stratec, Germany)^21^. DNA was extracted using ZymoBIOMICS^™^ DNA Kit (Zymo Research, USA) for 16S rRNA sequencing on the Illumina MiSeq platform^22^. CD4^+^, CD8^+^ T-cell counts, and HIV load (VL) were determined by flow cytometry and quantitative PCR, respectively^23^. Clinical data regarding ART, and BMI were retrieved from the CRF (clinical record form) and medical records.

### Sequence analysis

Paired end Illumina reads were checked for quality using FastQC^24^ and trimmed using Cutadapt^25^. The taxonomic classification and analysis of the trimmed reads were performed using dada2^26^ within Qiime2^27^ in combination with SILVAv132 database^28^. Alpha diversity of the samples was estimated using the R function *estimate_richness* in R package phyloseq (v1.30.0)^29^ and visualized using R package ggplot2 (v3.3.5)^30^. The diversity indices such as Observed, Shannon, and Simpson were performed to calculate the richness and diversity of the samples. The samples were clustered based on the distance method Bray–Curtis and visualized using non-metric multidimensional scaling (NMDS) ordination plots. The significance of the different factors on the beta-diversity were calculated based on PERMANOVA using vegan package (v2.5.7) (Adonis function). Linear discriminant analysis Effect Size (LEfSe) was employed to determine the significant microbial communities between the groups with LDA score > 2 and P < 0.05^31^ and visualized using R package ggplot2. Correlation analysis was performed using Spearman correlation method using R package psych (v2.2.3)^32^ and results were visualized using R package ggplot2 (v3.3.5).

### Ethics approval and consent to participate

The Swedish Ethical Review Authority (ID 2021-00,451, ID 2023-05,153-02) thoroughly examined and granted approval for the ethical permit, and every participant duly furnished written informed consent.

## Results

### Study participants

Among the 69 PLWH participants in this study, the median age was 54 (IQR, 45–62) years, the median duration on ART was 7 (IQR, 4–15) years, and the median BMI was 25 (IQR, 23–27) kg/m^2^. For the HC (n = 80), the median age was 53 (IQR, 34–66) years and the median BMI was 25.1 (IQR, 23–29) kg/m^2^. More than 90% of PLWH had less than 50 HIV RNA copies/mL at the microbiome collection. There were no significant differences between age, sex, and BMI between PLWH and HC (Table 1). The ART regimens included a backbone of NRTIs with either an INSTI (n = 56) or an NNRTI (n = 13), and PI (n = 2) as third drug. INSTI treated participants were either on dolutegravir (DTG, 75%) or bictegravir (BIC, 23%). The major modes of transmission (MSM vs. Heterosexual) were similarly distributed among those treated with INSTIs and NNRTIs.

**Table 1:** Baseline demographic and clinical characteristics of the study participants.

	PLWH (n = 69)			HC (n = 80)	P-value
	INSTI (n = 54)	NNRTI (n = 13)	PI* (n = 2)		
Sex, n (%)					
Man	35 (64)	6 (46)	0 (0)	47 (59)	1
Woman	19 (36)	7 (54)	2 (100)	33 (41)	
Age (years)	54 (45–62)	58 (44–63)	47 (44–49)	53 (34–66)	0.24
BMI (kg/m^2^)	25 (23–27)	24.9 (23–27)	28 (26–30)	25.1 (23–29)	0.76
Ethnicity, n(%)					
Caucasian	32 (58)	6 (47)	1 (50)	70 (88)	
Latin	1 (2)	1 (8)	0 (0)	1 (1)	
Asian	9 (17)	4 (30)	0 (0)	3 (4)	
Black	9 (17)	2 (15)	1 (50)	1 (1)	
Other/unknown	3 (6)	0 (0)	0 (0)	5 (6)	
Diet, n (%)					
Omnivorous	49 (91)	13 (100)	2 (100)	70 (88)	
Vegetarian	4 (7)	0 (0)	0 (0)	6 (8)	
Others/Unknown	1 (2)	0 (0)	0 (0)	4 (4)	
Duration of ART (years)	7 (4–15)	11 (8–16)	20 (19–22)	NA	0.16
Mode of transmission, n (%)					
Blood transfusions	1 (2)	1 (8)	0 (0)	NA	
Heterosexual	35 (64)	8 (61)	2 (100)		
Homosexual, bi-sexual	1 (2)	0 (0)	0 (0)		
IVDU	1 (2)	0 (0)	0 (0)		
MSM	15 (28)	4 (31)	0 (0)		
Perinatal	1 (2)	0 (0)	0 (0)		
CD4^+^ T-cell count	620 (280–730)	610 (460–698)	685 (623–748)	NA	0.71
CD4/CD8 ratio	0.89 (0.43–1.3)	1.33 (0.75–1.4)	1.02 (0.94–1.1)	NA	0.05
Nadir CD4^+^ T-cell count	280 (80–432)	220 (138–410)	338 (254–421)	NA	0.71
HIV RNA < 50 (c/ml) at baseline (%)^#^	87	92	100	NA	0.39

Table 1 the Mann–Whitney U test was applied to compare the continuous variables and Fisher’s exact test to analyze the categorical variables. All baseline characteristics are illustrated as median (inter quartile range) and demographic characters are illustrated as n (%). *Denotes that these individuals were not included in the calculation of p-values. ^#^Denotes that out of 69 individuals in PLWH, 7 individuals had HIV RNA level > 50 (c/mL) with range 53–132.

### Lower bacterial diversity and enrichment of pathobionts in PLWH compared to HC

**Figure 1:**
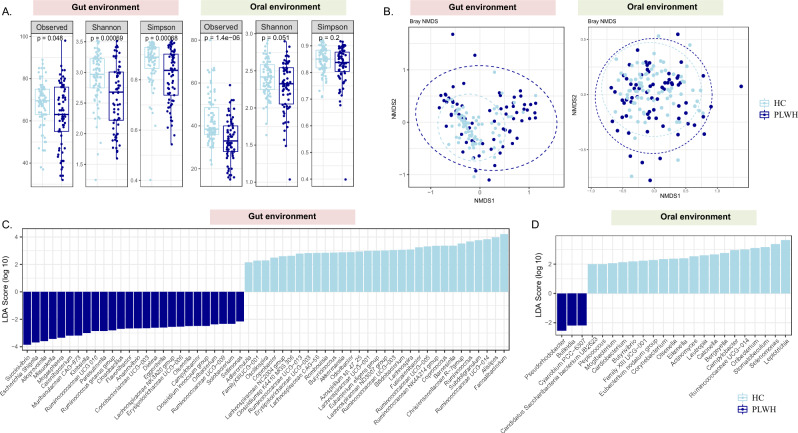
Alpha diversity and compositional changes in the gut and oral microbiome between Healthy Controls (HC, n = 80) and People living with HIV (PLWH, n = 69). (**A**) Boxplots showing the differences in the alpha diversity indices within the HC and PLWH in the gut and oral environment (**B**) NMDS plot illustrating the changes of beta diversity within the HC and PLWH in the gut and oral environment. Linear discriminant analysis effect size (LEfSe) analysis at the genus level showing the differentially abundant microbiota between HC and PLWH in the (**C**) gut and (**D**) oral samples, respectively.

PLWH showed significantly lower alpha diversity, particularly richness, compared to HC in both fecal (Observed p = 0.048, Shannon, p = 0.001, Simpson p = 0.001) and oral samples (Observed p < 0.0001, Shannon, p = 0.051) (Fig. 1A). Additionally, there were also significant differences in the beta diversity between these two groups (Fig. 1B, p = 0.001), with distinct clustering patterns in both gut and oral environments (Fig. 1B). A total of 258 bacterial taxa were detected in the entire cohort including both fecal and oral samples with several significant differences in microbial composition between PLWH and HC. For the fecal samples, *Klebsiella* (p = 0.046), *Succinivibrio* (p = 0.014), *Escherichia-Shigella* (p < 0.0001), *Cloacibacillus* (p = 0.03) and *Ruminococcus gnavus* group (p = 0.02) were significantly enriched in PLWH, whereas *Faecalibacterium* (p < 0.0001), *Ruminococcus* (p = 0.001), *Lachnospira* (p = 0.02) and *Bifidobacterium* (p = 0.004) were significantly more abundant in the HC (Fig. 1C). In the oral samples, *Pseudorhodobacter* (p = 0.01) and *Bulleidia* (p = 0.018) were significantly more abundant in PLWH and *Leptotrichia* (p = 0.01) in HC (Fig. 1D).

### Alterations in the microbial compositions in PLWH based on immune status and time on antiretroviral therapy

**Figure 2:**
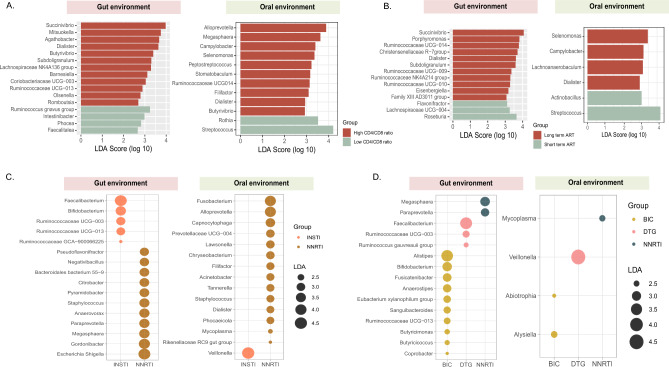
Differences in abundance of bacterial taxa evaluated using Linear discriminant effect size (LEfSe) analysis in both gut and oral environment in (**A**) Individuals with high (n = 39) and low CD4/CD8 ratio (n = 30), (**B**) individuals who received long-term ART (n = 49) or short-term ART (n = 20), (**C**) PLWH on NNRTI (n = 13) and INSTI (n = 53) and (**D**) subjects under different INSTI drug regimens (DTG, n = 41) (BIC, n = 12) and NNRTI treatment (n = 13). NNRTI: non-nucleoside reverse transcriptase inhibitors, INSTI: integrase strand transfer inhibitors.

PLWH were further stratified for three parameters into two groups based on CD4^+^ T-cell count (< / ≥ 350 cells/µL), CD4^+^ Nadir (< / ≥ 200 cells/µL), CD4/CD8 ratio (< / ≥ 0.79), and time on ART (< / ≥ 5 years). There were no differences observed in the alpha diversity indices between the individuals belonging to the different groups defined by above-mentioned variables (data not shown). However, individuals with high CD4/CD8 ratio showed higher abundance of *Dialister* (p = 0.03), *Agathobacter* (p = 0.03), *Succinivibrio* (p = 0.03), and *Butyrivibrio* (p = 0.01) in the gut environment and *Dialister* (p = 0.01), *Alloprevotella* (p = 0.03) and *Megasphaera* (p = 0.02) in the oral environment. Conversely, individuals with low CD4/CD8 ratio were significantly enriched with *Ruminococcus gnavus* group (p = 0.03) in the gut environment and *Streptococcus* (p = 0.04) and *Rothia* (p = 0.03) in the oral environment (Fig. 2A).

Furthermore, *Phascolarctobacterium* (p = 0.03) was significantly more abundant in individuals with low CD4^+^ T-cell count whereas *Dialister* (p = 0.047), and certain members of Ruminicoccacea family, such as *Ruminococcacea UCG-002* (p = 0.04) and *Ruminococcacea UCG-013* (p = 0.02) were increased in individuals with high CD4^+^ T-cell count in the fecal samples (Fig S1 A). Moreover, the oral microbiome also showed enrichment of *Dialister* (p = 0.016) and *Megasphaera* (p = 0.003) in individuals with high CD4^+^ T-cell count (Fig S1 B). Similarly, when stratified based on their nadir CD4^+^ T-cell count, in the gut environment we observed a significant abundance of *Succinivibrio* (p = 0.03) and *Dialister* (p = 0.04) in PLWH with high nadir CD4 ^+^ T-cell count and *Bacteriodes* in PLWH with low nadir CD4 ^+^ T-cell. In the oral environment, we found a significant increase in *Megasphaera* (p = 0.003) and *Dialister* (p = 0.02) in PLWH with high nadir CD4^+^ T-cell count and *Neisseria* (p = 0.02) in PLWH with low nadir CD4 ^+^ T-cell count (data not shown).

PLWH on ART for more than 5 years had increased numbers of *Succinivibrio* (p = 0.024), *Christensenellaceae R-7* group (p = 0.015), and *Dialister* (p = 0.04) in the fecal samples, while *Roseburia* (p = 0.03) was significantly more abundant in PLWH on short-term ART (Fig. 2B). Moreover, in oral samples, individuals on longer duration of ART showed an abundance of *Selenomonas* (p = 0.016), *Camphylobacter* (p = 0.04) and *Dialister* (p = 001), which were also observed in individuals with high CD4/CD8 ratio. Likewise, individuals on short-term ART and with low CD4/CD8 ratio showed an abundance of *Streptococcus* (p = 0.04).

### Differences in the gut microbiome associated with different treatment regimens

To explore the influence of the INSTIs and NNRTIs on microbiome, we stratified PLWH based on their drug regimen. In the fecal samples, *Faecalibacterium* (p = 0.02) and *Bifidobacterium* (p = 0.04) were significantly more abundant in PLWH on INSTIs, while *Escherichia-Shigella* (p = 0.04), *Gordonibacter* (p = 0.04), *Megasphaera* (p = 0.01), and *(*p = 0.04) were enriched in PLWH on NNRTIs (Fig. 2C). In the oral samples, we found higher abundance of *Veillonella* (p = 0.006) in INSTI-treated individuals and significant enrichment of *Fusobacterium* (p = 0.02), *Alloprevotella* (p = 0.03), *Staphylococcus* (p = 0.04), and *Dialister* (p = 0.03) in PLWH on NNRTI-treatment.

We further analyzed PLWH on dolutegravir (DTG) or bictegravir (BIC) and compared them to NNRTI. In the gut environment, a higher abundance of *Bifidobacterium* (p = 0.02), Anerostipes (p = 0.03), *Butyricimonas* (p = 0.04) and *Butyricicoccus* (p = 0.045) was observed in BIC-treated individuals, *Faecalibacterium* (p = 0.04) and *Ruminococcus gauvreauii* group (p = 0.03) in DTG-treated individuals and *Megasphaera* (p = 0.02) in NNRTI-treatment recipents (Fig. 2D). Conversely, in the oral environment we observed higher abundance of *Alysiella* (p = 0.003), *Veillonella* (p = 0.02), and *Mycoplasma* (p = 0.03) in BIC-, DTG-, and NNRTI-treated PLWH, respectively.

Since mode of transmission (MOT) has been identified as one of the factors influencing microbiome in PLWH^3,13^, we stratified the individuals with different MOT (MSM vs Heterosexuals) into separate treatment groups. The same microbiome markers were not associated with MOT groups but varied within treatment groups (data not shown). This implies that MOT was not the major driver of microbiome changes in our cohort.

### Relationship between gut microbiota composition and BMI

**Figure 3:**
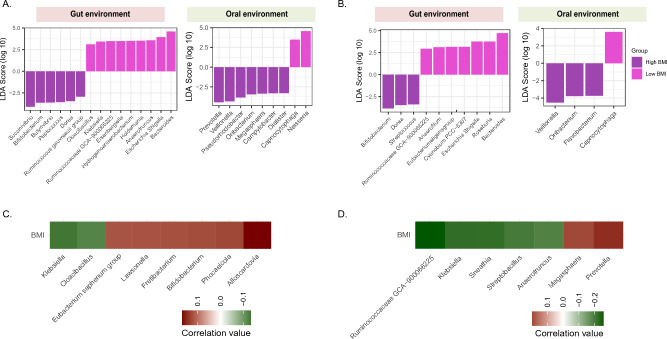
Differences in the gut and oral microbiome in PLWH, further divided into two groups based on BMI. Linear discriminant analysis effect size (LEfSe) analysis showing the significant microbial organisms between individuals with high and low BMI (< / ≥ 25 kg/m^2^) in the gut and oral environment: differences (**A**) in PLWH (high BMI n = 37, low BMI, n = 32), and (**B**) in PLWH treated with DTG (high BMI n = 24, low BMI n = 17). Spearman correlations showing the association between BMI and microbial composition at the genus level within the gut and oral environment in the (**C**) whole cohort and (**D**) PLWH.

Based on the potential clinical association between INSTI treatment and weight gain reported in few studies^19,20^, we further explored the link between microbiome, ART, and BMI in our cohort. In the gut microbiome of PLWH, *Succinivibrio* (p = 0.045), *Dorea* (p = 0.004), and *Bifidobacterium* (p = 0.03) were significantly higher in individuals with high BMI (> 25) and *Escherichia-Shigella* (p = 0.01), *Bacteroides* (p = 0.04) and *Klebsiella* (p = 0.03) were enriched in group with low BMI (< 25). In oral samples, we observed higher abundance of *Prevotella* (p = 0.02), *Dialister* (p = 0.004), and *Veillonella* (p = 0.01) in PLWH with overweight and *Neisseria* (p = 0.03) in PLWH with low BMI (Fig. 3A, B). Similar microbial signatures were also observed in individuals with high and low BMI belonging to the whole cohort (PLWH and HC) in both oral and gut samples (Fig S2). These signatures were most likely shaped by PLWH status, since stratifying the HC group into high and low BMI have not revealed similar associations.

DTG has been primarily associated with visceral fat accumulation^33^. As nearly 70% of all PLWH were treated with DTG, we sub-categorized DTG-treated individuals based on low and high BMI. In the fecal samples *Bifidobacterium* (p = 0.01), *Dorea* (p = 0.03), and *Streptococcus* (p = 0.01) were significantly more abundant in people with high BMI, while *Bacteroides* (p = 0.047) and *Escherichia-Shigella* (p = 0.045) were more abundant in people with low BMI.

We further investigated correlations between BMI and abundance of bacterial taxa in the whole cohort. We observed that *Bifidobacterium* (p = 0.04) was positively correlated with BMI and *Klebsiella* (p = 0.03), *Escherichia-Shigella* (p = 0.05)*,* and *Cloacibacillus* (p = 0.046) were negatively correlated with BMI, in both oral and gut environment. In PLWH, positive correlation between *Prevotella* (p = 0.02), *Dialister* (p = 0.05), *Megasphaera* (p = 0.04)*, Bifidobacterium* (p = 0.058) and BMI and negative correlation of *Klebsiella* (p = 0.02), *Escherichia-Shigella* and BMI were found (Fig. 3C, D).

### Effect of DTG on the gut and oral microbiota

We conducted a more in-depth analysis of the associations between the microbiome and several clinical factors, such as age, duration of treatment, and CD4^+^ T-cell counts in PLWH on DTG. In the gut milieu, alpha diversity was lower in the younger individuals (18–39 years) compared to the elderly (> 60 years) (p < 0.01, Fig S3 A). We also found significant differences in beta-diversity among the age groups (p = 0.05) (Fig S3 B). At the genus level, younger individuals displayed a significantly greater abundance of *Lachnospira* (p = 0.04) and *Eggerthella* (p < 0.0001), while elderly individuals harbored a higher abundance of *Coprococcus* (p = 0.01) and *Dorea* (p = 0.01) (Fig S3 C). However, for oral samples, we found no significant differences in alpha and beta diversity between the age groups (Fig S3 D, E). We also found that *Kingella* was significantly abundant in younger individuals and *Leptotrichia* and *Ruminococcaceae UCG-004* were abundant in the middle-aged group (40–59 years) (Fig S3 F).

Moreover, in the DTG-treated group, PLWH with longer treatment duration exhibited significantly higher alpha diversity indices in fecal microbiome compared to those on short-term ART (Fig S4 A), with a higher abundance of *Succinivibrio* (p = 0.034) (Fig S4 B). On the other hand, the alpha diversity hasn’t changed significantly in between the individuals during longitudinal follow-up and short-term follow-up in the oral compartment (Fig S4 C), although the saliva samples of individuals under long-term ART had a higher prevalence of *Dialister* (p = 0.04) (Fig S4 D).

Additionally, gut microbiome richness was increased in PLWH on DTG and higher CD4^+^ T-cell counts compared to those with lower immune status (Observed p = 0.014, Fig S5 A). We observed an enrichment of *Fusobacterium* (p = 0.05), *Ruminococcus gnavus* group (p = 0.02), and *Lachnoclostridium* (p = 0.01) in individuals with lower CD4^+^ T-cell counts, whereas *Dialister* (p = 0.01), *Ruminococcus* (p = 0.004), and *Agathobacter* (p = 0.02) were more abundant in those with higher CD4^+^ T-cell counts (Fig S5 B). Conversely, oral microbiome richness and diversity was higher in individuals with low CD4^+^ T-cell counts compared to that of individuals with higher counts (Simpson p = 0.046, Fig S5 C). As for oral samples, an enrichment of *Peptococcus* (p = 0.02), *Kingella* (p = 0.01), and *Paludibacteraceae* F0058 (p = 0.002) was noted in DTG-treated individuals with low CD4^+^ T-cell counts (Fig S5 D).

## Discussion

In our work, we investigated the shifts within the gut and oral microbiome of PLWH in relation to different ART components and immune status. Furthermore, we explored the correlation between microbiome, antiretroviral treatments, and BMI, since weight gain is reported as a potential adverse outcome associated with certain ART regimens^19,20,33–35^.

Initially, we observed that PLWH had significantly lower richness and different bacterial composition compared to HC, in both the oral and gut environments. These differences were present even if the PLWH had been on efficient long-term ART with sustained high CD4^+^ T-cell counts and undetectable HIV RNA. Specifically, we noted an enrichment of *Bifidobacterium, Lachnospira, Akkermansia*, and *Faecalibacterium* in the gut microbiome of HCs. On the contrary, there were increased levels of potentially pathogenic bacteria such as *Succinivibrio, Megasphaera, Klebsiella, Escherichia-Shigella*, and *Ruminococcus gnavus* group in PLWH. Moreover, bacterium such as *Bifidobacterium* are known for their probiotic qualities and play a critical role in the effective functioning of the immune system^36^. Similarly, *Faecalibacterium* and *Akkermansia* possess anti-inflammatory properties and are instrumental in governing immune activation, host metabolism, and the preservation of gut barrier integrity^37^. Additionally, *Lachnospira* is known to produce beneficial metabolites conducive to gut health^38^. Our findings suggest that *Bifidobacterium, Lachnospira, Akkermansia*, and *Faecalibacterium* may serve as markers of a healthy gut. Conversely, *Escherichia-Shigella and Klebsiella*, whilst common gut commensals, have the potential to become opportunistic pathogens in individuals with compromised immune system ^39^. *Escherichia-Shigella* produces various proinflammatory components such as lipopolysaccharide and peptidoglycans which could contribute to excessive intestinal inflammation^40^. The presence of these bacteria suggests the increased abundance of certain pathobionts in the gut of PLWH. In the oral environment, we found that *Bulleidia* was enriched in PLWH, which is more frequently observed in individuals with periodontitis^41^. Conversely, bacteria such as *Leptotrichia* and *Selenomonas* were increased in HC. Studies have shown that both these taxa are a part of the normal oral microbiome^42^.

We did not observe any significant microbiome diversity changes based on the immune status and length of ART. Nevertheless, the gut bacterial communities showed an enrichment of *Succinivibrio* in the PLWH with high CD4/CD8 ratio and long-term ART. Several earlier studies have reported that higher abundance of *Succinivibrio* in not only PLWH under ART^3,5,43^ but also in untreated HIV positive elite controllers^43^. We also observed the enrichment of *Ruminococcus gnavus* in PLWH with low CD4/CD8 ratio, a bacterium associated with inflammatory bowel disease and known to produce imidazole propionate^44,45^. Imidazole propionate was recently linked to type 2 diabetes and cardiovascular risk in the general population^46^. The enrichment of *Ruminococuus gnavus* in individuals with low CD4/CD8 ratio may reflect the proinflammatory state associated with increased comorbidity risk present in these individuals^47,48^. Furthermore, in the oral environment we found an abundance of *Megasphaera* in PLWH with high CD4^+^ T-cell count and high CD4/CD8 ratio, as previously reported^49^. *Streptococcus* was also significantly enriched in PLWH who were on short term ART and with low CD4/CD8 ratio. Likewise, several recent studies which explored the salivary microbiome, showed that the abundance of *Streptococcus* was increased in PLWH and associated with systemic inflammation^15,50,51^.

Intriguingly, we found an enrichment of *Bifidobacterium* and *Faecalibacterium* within the gut microbiome of individuals treated with INSTIs, a fact noteworthy even considering previous studies that reported an increase of *Faecalibacterium* in ART treated individuals^11,52^. It is plausible to speculate that the presence of these taxa in INSTI-treated individuals, in contrast to those treated with NNRTIs, could reflect their superior immune status or immune reconstitution, as previously proposed^53^. However, this association was not present in our study, suggesting the need for future prospective studies to further investigate this hypothesis. In the oral samples the genus of *Veillonella* was increased in INSTI-treated individuals and consequently in PLWH on DTG. *Veillonella* is an anaerobic bacterium, commonly found in the microbiota of the mouth, gut, and vagina. It has the ability to ferment lactic acid and use it as a primary source of energy. Alterations of *Veillonella* species in the gut microbiome have been reported in PLWH but not in connection to INSTI treatment^54^. In contrast, in the NNRTI-treated group, we observed the presence of *Gordoniobacter, Megasphaera* and *Fusobacterium* in the gut and oral environment, respectively. Some *Megasphaera* species have the ability to ferment sugars and organic acids, including lactate, into volatile fatty acids such as butyrate, propionate, and acetate^55^. These short-chain fatty acids are essential for maintaining gut health as they serve as an energy source for colon cells and have anti-inflammatory properties^56^.

Previous studies have demonstrated a link between ART regimens, specifically INSTIs, and obesity^34,35,57,58^. In our cohort, we identified an increased abundance of *Bifidobacterium* and *Dorea* in individuals with high BMI. These findings are particularly interesting since earlier studies have suggested an inverse association between *Bifidobacterium* and obesity, indicating a potential protective role of *Bifidobacterium* in weight gain, fat distribution and impaired glycemic control^59^. However, certain clinical studies have found an enrichment of *Bifidobacterium* in PLWH with high BMI, indicating the complexity of interactions within the microbiota of PLWH^60^. Conversely, studies have shown a higher prevalence of *Dorea* in HIV infected individuals with metabolic syndrome^61^. The presence of *Dorea* has been associated with insulin secretion and fasting blood glucose levels, implying its potential involvement in the progression of type 2 diabetes in overweight and obese individuals^62^. Intriguingly, our study also found an enrichment of proinflammatory pathobionts, such as *Klebsiella and Escherichia-Shigella*, in individuals with lower BMI. We observed a negative correlation between BMI and the presence of *Klebsiella, Escherichia-Shigella*, and *Cloacibacillus*, whereas a positive correlation was noted between BMI and the abundance of *Bifidobacterium* and *Prevotella* particularly in PLWH. In the oral microbiome of PLWH with overweight, there was a noticeable enrichment of *Prevotella and Veillonella*. Conversely, those subjects with a lower BMI exhibited an increased presence of *Neisseria*. Research within the field of dental medicine has earlier suggested that the oral microbiome of individuals with obesity is characterized by an escalation of traditional periodontal pathogens. However, the precise mechanisms driving these alterations remain to be elucidated^63^.

We acknowledge some limitations of our study. Since we have employed the 16S rRNA gene sequencing method, due to the homology between the sequences, 16S rRNA sequencing technique may not be able to distinguish related bacterial species^64^. Furthermore, we could not evaluate functional bacterial pathways thereby preventing a deeper understanding of the microbiome's metabolic activities and interactions and further discriminating cause-and-effect relationship. Another limitation of our study is the use of BMI as a marker for weight gain. While BMI is a surrogate marker for weight gain^65^, additional measurements such as waist circumference can provide a more precise assessment of obesity. Lastly, we only collected basic dietary information from participants; other factors, such as the types of nutrients consumed and lifestyle habits, were not recorded. Despite these limitations, our study involved the incorporation of a good number of participants, irrespective of our patient exclusion criteria. In addition, we ensured that the HC were carefully matched to PLWH in age groups and gender, strengthening the validity of our comparative analysis.

Overall, our study shows that there are associations between several components of fecal and oral microbiome in relation to different ART regimens and BMI in PLWH. We evidently demonstrate that the bacterial diversity was higher in HC compared to PLWH in both the gut and oral environment. We also observed several microbial markers associated with different ART treatments. Notably, the most prominent feature was the abundance of *Bifidobacterium* and *Faecalibacterium* in INSTI-treated individuals in the gut environment and *Veillonella* in the oral environment. The varying correlation of certain bacterial genera with BMI in both HC and PLWH might reflect how different health conditions, immune status, and host metabolism can influence the composition of the gut microbiota. Further research in this field will be valuable for better understanding of these cause-and-effect relationships and may provide insights for potential therapeutic interventions to optimize the gut microbiota in the context of obesity and HIV infection.

### Supplementary Information

Supplementary Figures.

## Data Availability

The data resulting from this study, including both the gut and oral metadata and the raw 16S rRNA sequences, have been archived in the NCBI SRA database under project numbers PRJNA902956 and PRJNA900274.
