# Supplementary material for: Exploring the interplay between antiretroviral therapy and the gut-oral microbiome axis in people living with HIV
Source: Sci Rep. 2024 Aug 1;14:17820. doi: 10.1038/s41598-024-68479-4 (PMC11294597; doi:10.1038/s41598-024-68479-4)
Supplement: Supplementary file 1 — Supplementary Figures. [file 41598_2024_68479_MOESM1_ESM.pdf]

**Supplementary figure legends**

Fig S1: Significant changes in the microbial composition assessed using Linear discriminant effect size (LEfSe) analysis between patients with high and low $CD4^+$ T cell response in the (A) gut and (B) oral environment. High $CD4^+$ T-cell count, n=48; low $CD4^+$ T-cell count, n=21.

Fig S2: Linear discriminant effect size (LEfSe) analysis showing significant differences in bacterial taxa in the whole cohort in individuals with high and low BMI in the (A) gut and (B) oral environment. High BMI, n=81; low BMI, n=55.

Fig S3: Changes in the alpha diversity, beta diversity, and composition using Linear discriminant effect size (LEfSe) analysis in DTG-treated patients in different age groups (18-39, 40-59 and >60 years) in (A-C) the gut environment and (D-F) the oral environment. 18-39 years, n=8; 40-59 years, n=19; >60 years, n=14.

Fig S4: Changes in the alpha diversity and composition using Linear discriminant effect size (LEfSe) analysis in DTG-treated patients divided into groups based on the length of antiretroviral therapy (</≥5 years) in (A-B) the gut environment and (C-D) the oral environment. ≥5 years, n=26; <5years, n=15.

Fig S5: Changes in the alpha diversity and composition using Linear discriminant effect size (LEfSe) analysis in DTG-treated patients divided into groups based on $CD4^+$ T-cell count (</≥350) in (A-B) the gut environment and (C-D) the oral environment. ≥350 $CD4^+$ T-cell count, n=29; <350 $CD4^+$ T-cell count, n=12.

Fig S1

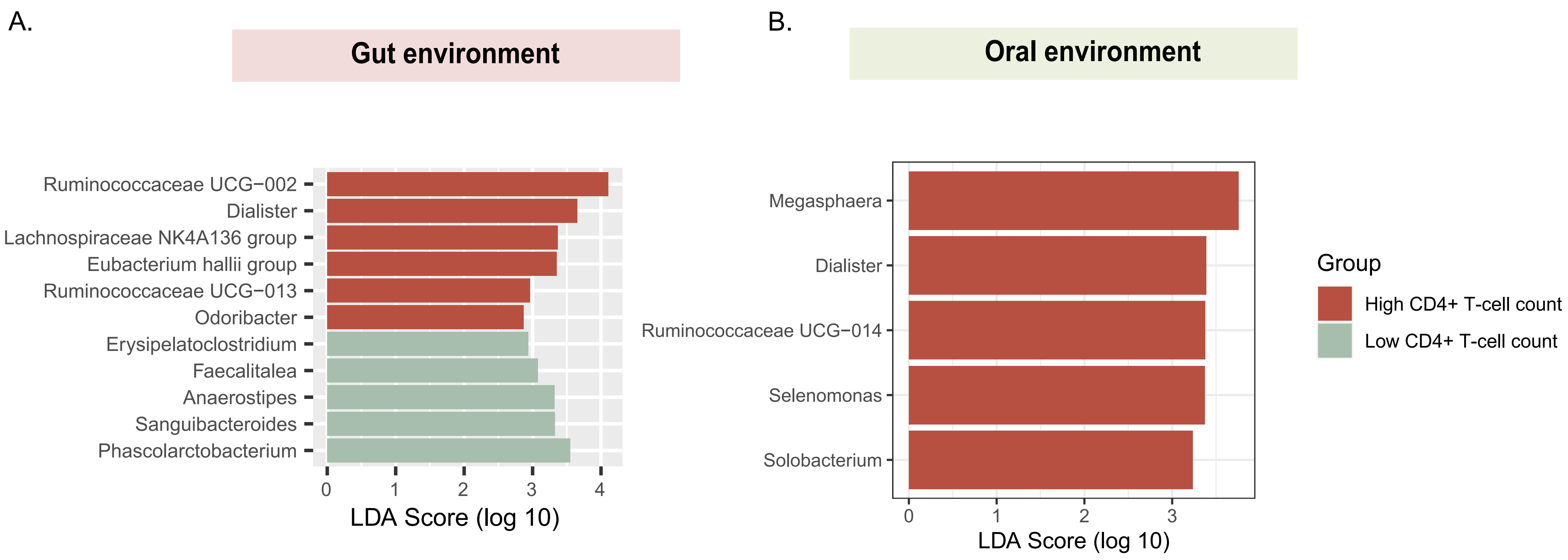
A.
Gut environment
Ruminococcaceae UCG−002
Dialister
Lachnospiraceae NK4A136 group
Eubacterium hallii group
Ruminococcaceae UCG−013
Odoribacter
Erysipelatoclostridium
Faecalitalea
Anaerostipes
Sanguibacteroides
Phascolarctobacterium
0
1
2
3
4
LDA Score (log 10)
B.
Oral environment
Megasphaera
Dialister
Ruminococcaceae UCG−014
Selenomonas
Solobacterium
0
1
2
3
LDA Score (log 10)
Group
High CD4+ T-cell count
Low CD4+ T-cell count

Fig S2

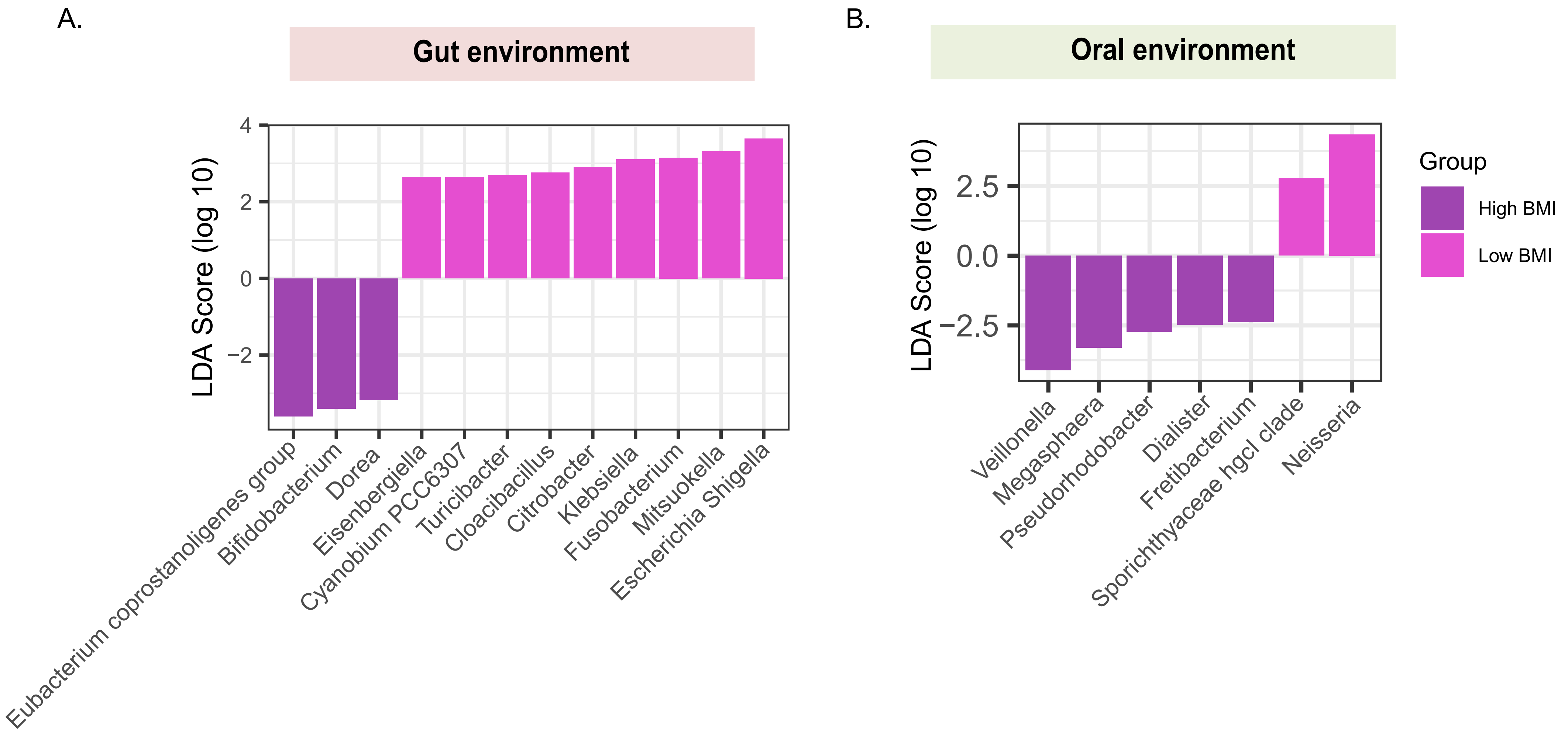
A.
Gut environment
LDA Score (log 10)
4
2
0
−2
Eubacterium coprostanoligenes group
Bifidobacterium
Dorea
Eisenbergiella
Cyanobium PCC6307
Turicibacter
Cloacibacillus
Citrobacter
Klebsiella
Fusobacterium
Mitsuokella
Escherichia Shigella
B.
Oral environment
LDA Score (log 10)
2.5
0.0
−2.5
Veillonella
Megasphaera
Pseudorhodobacter
Dialister
Fretibacterium
Sporichthyaceae hgcl clade
Neisseria
Group
High BMI
Low BMI

Fig S3

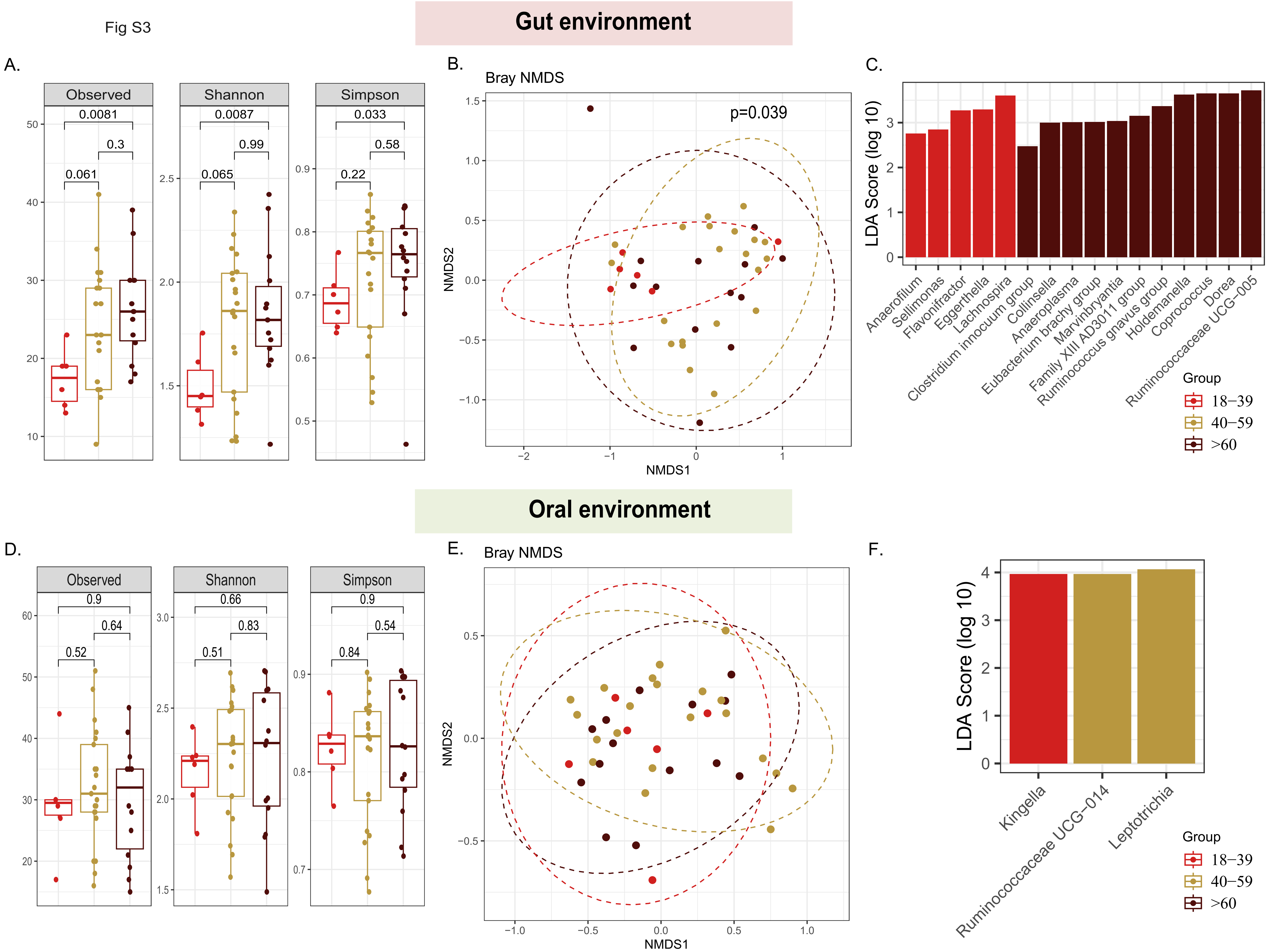
Gut environment
A.
Observed
Shannon
Simpson
0.0081
0.3
0.061
0.0087
0.99
0.065
0.033
0.58
0.22
B.
Bray NMDS
p=0.039
NMDS1
NMDS2
C.
LDA Score (log 10)
Anaerofilum
Sellimonas
Flavonifractor
Eggerthella
Lachnospira
Clostridium innocuum group
Collinsella
Anaeroplasma
Eubacterium brachy group
Marvinbryantia
Family XIII AD3011 group
Ruminococcus gnavus group
Holdemanella
Coprococcus
Dorea
Ruminococcaceae UCG−005
Group
18−39
40−59
>60
Oral environment
D.
Observed
Shannon
Simpson
0.9
0.64
0.52
0.66
0.83
0.51
0.9
0.54
0.84
E.
Bray NMDS
NMDS1
NMDS2
F.
LDA Score (log 10)
Kingella
Ruminococcaceae UCG−014
Leptotrichia
Group
18−39
40−59
>60

Fig S4

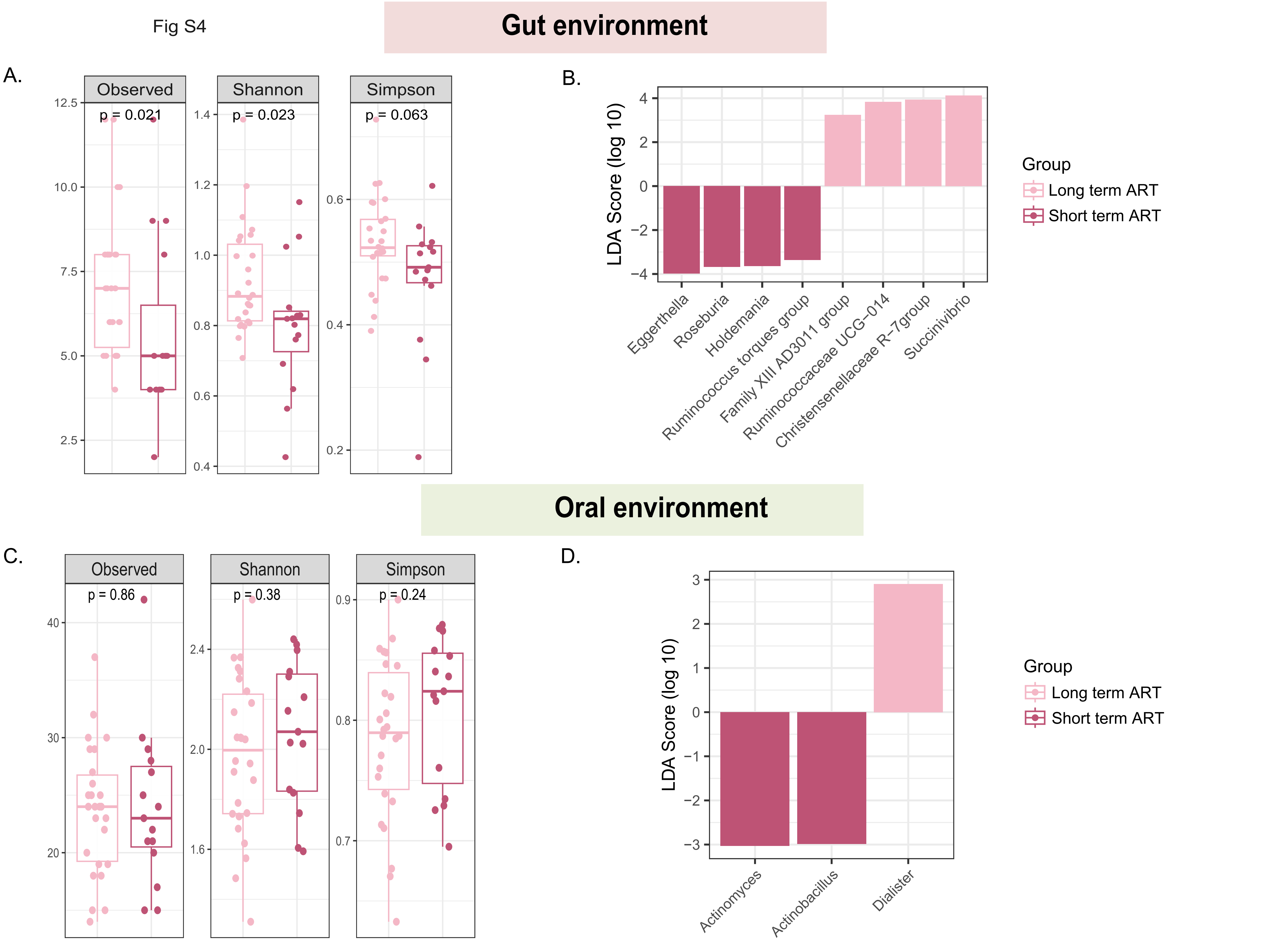
Gut environment
A.
Observed
Shannon
Simpson
p = 0.021
p = 0.023
p = 0.063
B.
LDA Score (log 10)
Eggerthella
Roseburia
Holdemania
Ruminococcus torques group
Family XIII AD3011 group
Ruminococcaceae UCG−014
Christensenellaceae R−7group
Succinivibrio
Group
Long term ART
Short term ART
Oral environment
C.
Observed
Shannon
Simpson
p = 0.86
p = 0.38
p = 0.24
D.
LDA Score (log 10)
Actinomyces
Actinobacillus
Dialister
Group
Long term ART
Short term ART

Fig S5

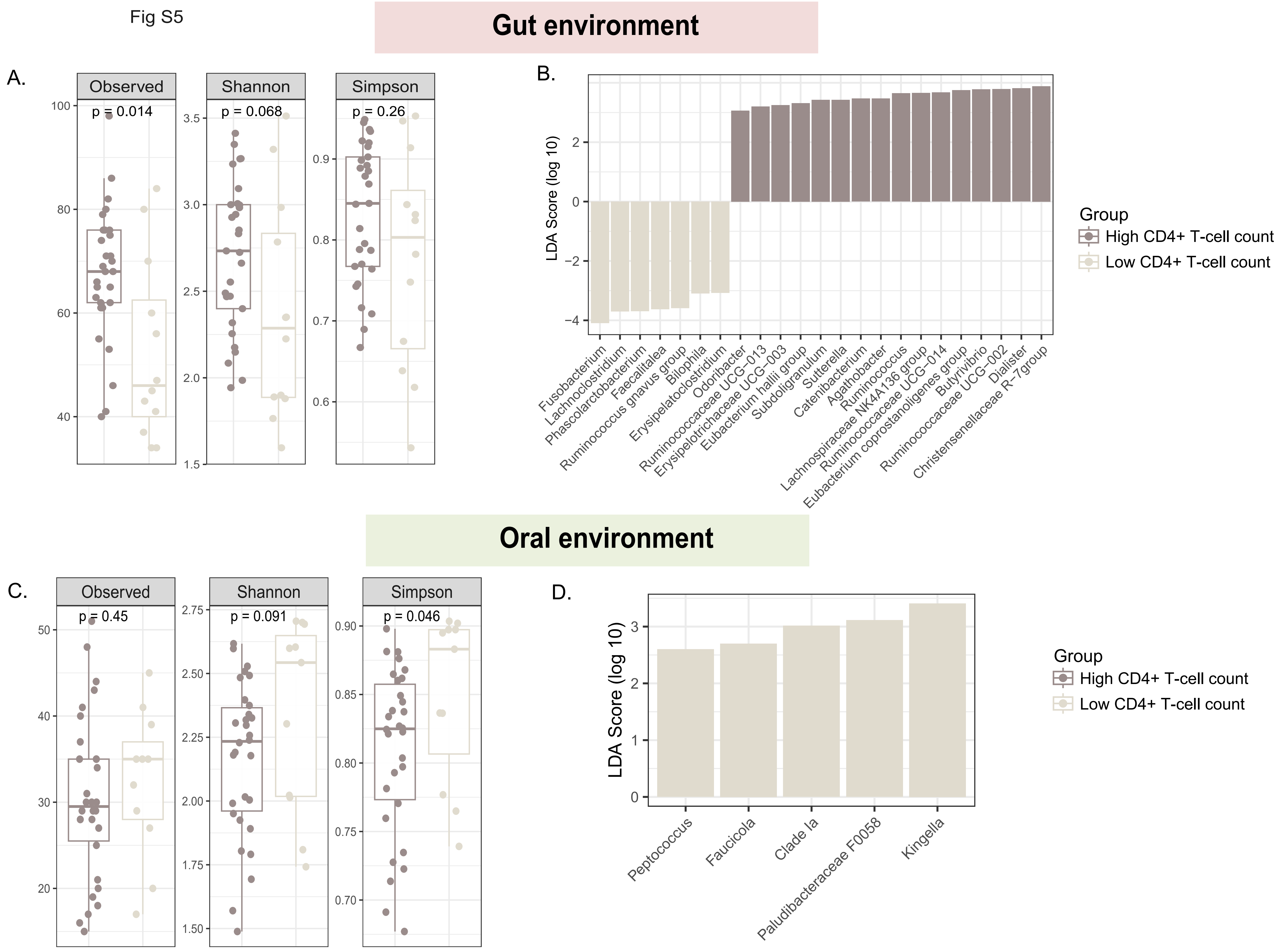

Gut environment
A.
Observed
Shannon
Simpson
p = 0.014
p = 0.068
p = 0.26
B.
LDA Score (log 10)
Fusobacterium
Lachnoclostridium
Phascolarctobacterium
Faecalitalea
Ruminococcus gnavus group
Bilophila
Erysipelatoclostridium
Odoribacter
Ruminococcaceae UCG−013
Erysipelotrichaceae UCG−003
Eubacterium hallii group
Subdoligranulum
Sutterella
Catenibacterium
Agathobacter
Ruminococcus
Lachnospiraceae NK4A136 group
Ruminococcaceae UCG−014
Eubacterium coprostanoligenes group
Butyrivibrio
Ruminococcaceae UCG−002
Dialister
Christensenellaceae R−7group
Group
High CD4+ T-cell count
Low CD4+ T-cell count
Oral environment
C.
Observed
Shannon
Simpson
p = 0.45
p = 0.091
p = 0.046
D.
LDA Score (log 10)
Peptococcus
Faucicola
Clade Ia
Paludibacteraceae F0058
Kingella
Group
High CD4+ T-cell count
Low CD4+ T-cell count
